# The Effect of Direct Oral Anticoagulant Therapy (DOACs) on oral surgical procedures: a systematic review

**DOI:** 10.1186/s12903-023-03427-8

**Published:** 2023-10-11

**Authors:** Ghassan Darwish

**Affiliations:** Department of Oral and Maxillofacial Surgery, School of Dentistry, King Abdulaziz University, 21589 Jeddah, Saudi Arabia

**Keywords:** Direct oral anticoagulants, Dental extraction, Oral surgery

## Abstract

**Background:**

Direct oral anticoagulants (DOACs) were developed to overcome the drawbacks of oral anticoagulants. However, not much has been discussed about the perioperative management of patients on DOACs during oral surgical procedures. Thus, we aim to determine the risk of perioperative and postoperative bleeding during oral surgical procedures in patients on DOACs.

**Methods:**

A detailed literature search was performed to find potentially relevant studies using the Cochrane Library, Clinical Key, ClinicalTrials.gov, Google Scholar, Ovid, ScienceDirect, and Scopus. Every article available for free in English literature for the past 10 years, between 2012 and 2022, was searched.

**Results:**

A total of 2792 abstracts were selected through a search strategy across various search engines. Based on inclusion and exclusion criteria, eleven clinical studies using DOACs as anticoagulants or studies comparing patients with and without DOACs under oral surgery procedures were found. The results were inconsistent and varied, with a few studies recommending DOAC administration with the bare minimum reported complications and others finding no statistically significant difference between discontinuation or continuation of drugs, especially across basic dental procedures.

**Conclusion:**

Within the limitations of the study, it can be concluded that minor oral surgical procedures are safe for patients on DOAC therapy. However, the continuation or discontinuation of DOACs in patients undergoing oral surgical procedures remains controversial and requires further studies to extrapolate the results.

**Supplementary Information:**

The online version contains supplementary material available at 10.1186/s12903-023-03427-8.

## Background

Anticoagulants have become the predominant form of treatment in contemporary medicine. They are commonly recommended to lower the risk of thromboembolism in subjects with a history of angina, atherosclerosis, atrial fibrillation, cerebrovascular accidents, ischemic heart disease, myocardial infarction, and pulmonary embolism, thereby preventing the incidence of life-threatening events [1–4].

Oral anticoagulants include heparin, warfarin, and direct or new oral anticoagulants (DOACs or NOACs). Heparin is administered intravenously and interfers with the thrombin-antithrombin pathways, reducing fibrin formation [5]. Whereas warfarin, the 4-hydroxycoumarin derivative, is administered orally, it is widely used as the standard oral anticoagulant therapy (OAT) drug [2]. They are also commonly prescribed to elderly patients who may be required to undergo oral surgical procedures such as dental extractions, periodontal surgeries, alveoloplasty, dental implant surgery, and other pre-prosthetic surgeries for oral rehabilitation. However, the major drawback of oral anticoagulant therapy is the higher risk of hemorrhage after injuries or any surgical procedure [6]. Due to the increased risk of postoperative bleeding or oral hematoma in patients on anticoagulant therapy, performing surgical procedures in such patients becomes a significant concern for a dentist [7]. According to a study by Bump et al., fear of hemorrhages may elevate stress levels in cardiac patients, which may induce fibrinolytic activity [8].

To limit the risk of perioperative and postoperative bleeding associated with OAT, discontinuing anticoagulant treatment 2–3 days before oral surgical procedures is recommended [9, 10]. However, several studies suggest that discontinuing OAT for a short time may not be sufficient to stop perioperative complications and increase thromboembolic risk [2, 6, 11–15].

Recently, DOACs have been developed to overcome the drawbacks of OAT. DOACs are categorized into Xa inhibitors and direct thrombin inhibitors. Among Xa inhibitors, Rivaroxaban acts rapidly by inhibiting the Xa activity of the prothrombinase complex within 2.5–4 h [16]. Direct thrombin inhibitors such as Dabigatran inhibit the action of factor IIa in the coagulation pathway within 0.5–4 h [5, 7, 16, 17]. DOACs are known to have fewer drug-to-drug or drug-food interactions and a wide range of therapeutic windows that improve their safety. DOACs are more predictable than warfarin because of their simplified pharmacodynamics. They are more convenient, as they do not require periodic monitoring and are prescribed at fixed doses [18–20]. Besides, DOACs have gained popularity among cardiologists as the drug of choice to prevent stroke and thromboembolism [21, 22]. Shah et al., in their study, reported a significant reduction in transient ischemic attacks and systemic emboli events with Rivaroxaban compared to warfarin [23]. In another study by Costantinides et al., 1.6% of the risk of bleeding was observed with Apixaban compared to 1.9% with warfarin during dental extractions, colonoscopy, and ophthalmologic surgery [24].

Little is known about the perioperative management of patients taking DOACs during oral surgical procedures. This systematic review aims to determine the risk of perioperative and postoperative bleeding during oral surgical procedures in patients on DOACs. We also aim to assist dental professionals in making an informed clinical decision about continuing or discontinuing the DOACs before oral surgical procedures.

## Materials and methods

**Fig. 1:**
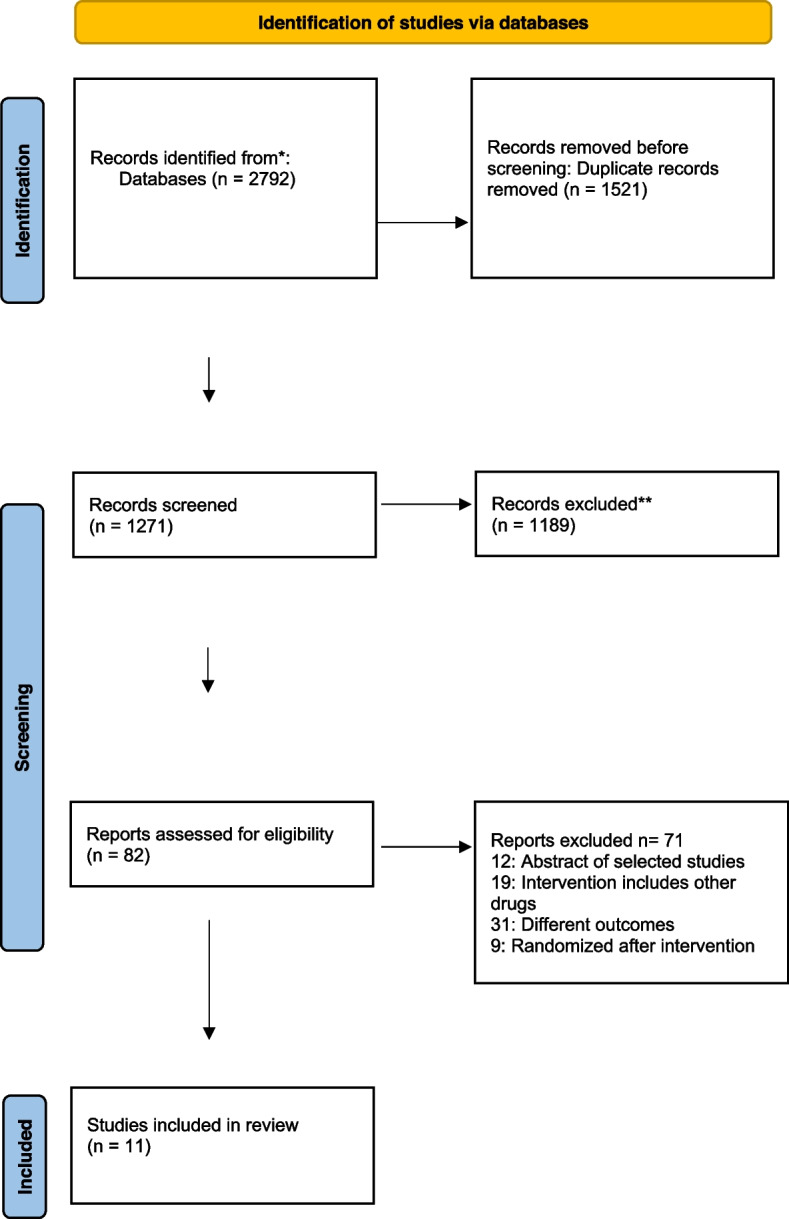
Prisma flow chart diagram

This systematic review was developed according to the Preferred Reporting Items for Systematic Review and Meta-Analyses (PRISMA) statement, as shown in Fig. 1. The Patient, Intervention, Comparison, and Outcome (PICO) format was used to find the focused primary question, which was “To determine the risk of perioperative and postoperative bleeding during oral surgical procedures in patients on DOACs”.

A detailed electronic literature search was performed to identify potentially relevant studies using the Cochrane Library, Clinical Key, ClinicalTrials.gov, Google Scholar, Ovid, ScienceDirect, and Scopus databases. Combined MeSH and accessible text search terms aimed to identify all anticoagulant agents, including synonyms, related terms, and variants, parenteral agents, “direct oral anticoagulants”, “oral surgery”, “dental extraction”, and “dental implants”, using PubMed and PubMed Central. Every article available for free in English literature for the past 10 years, between 2012 and 2022, was searched. Additionally, all related articles were scanned. Efforts were made to identify unpublished studies, especially the ones identified only in abstracts, but we could not reach the authors as not all details were available.

### Inclusion criteria

All prospective, retrospective, cohort, and RCTs (randomized-control trials) or studies comparing the patients on DOACs with patients without any anticoagulants that underwent oral surgical procedures such as extraction, periodontal surgery, dental implant surgery, pre-prosthetic surgeries, or alveoloplasties were included in our study. Studies identified for inclusion in the previous systematic review were reviewed and considered for inclusion in the data synthesis.

### Exclusion criteria

Case reports, case series, and literature reviews were excluded from our study.

### Study selection criteria

The outcome parameters on which we focused mainly were preoperative and postoperative bleeding, local hemostasis, delayed bleeding due to DOACs, discontinuation of DOACs during the procedure, and comparison with patients without anticoagulants. Based on our inclusion and exclusion criteria, all the titles and abstracts of the articles searched were scrutinized based on their relevance and eligibility for inclusion. Full texts were reviewed according to the inclusion criteria, and articles were finally selected for systematic review. Two reviewers screened independently and reviewed titles and abstracts for inclusion eligibility. They manually searched the references involving human subjects to identify relevant studies according to inclusion criteria. The same reviewers independently categorized the data, and the same two reviewers evaluated the data and results of the studies included in this assessment.

### Outcome measures

The primary outcome was the frequency of postoperative bleeding, defined as any bleeding occurring immediately after surgical procedures or up to 7 days after surgery. The primary independent variable was the type of anticoagulants taken by the patients; the secondary independent variable was the postoperative bleeding event.

### Quality assessment

For the clinical studies, the Newcastle–Ottawa Scale (NOS) was used to assess the quality of nonrandomized studies, as shown in Tables 1 and 2 [25].

**Table 1:** Newcastle Ottawa bias assessment for case–control studies

**Selection**					**Comparability**	**Outcome**			
**Authors & Year**	**Is the study Definition Adequate? (1)**	**Representativeness of the cases (1)**	**Selection of Controls (1)**	**Definition of Controls (1)**	**Comparability of samples and controls (2)**	**Assessment of exposure (1)**	**Same method of ascertainment for all samples (1)**	**No response rate (1)**	**Total quality score (9)**
**Miclotte et al.,2016**	1	1	1	1	2	1	1	1	9
**Gomez-moreno et al., 2018**	1	1	1	1	2	1	1	1	9
**Cappare et al.,** **2022**	1	1	1	1	2	1	1	1	9

**Table 2:** Newcastle Ottawa bias assessment for cohort studies

Selection					Comparability	Outcomes			
**Authors & year**	**Representativeness of the exposed cohort (1)**	**Selection of the non-exposed cohort (1)**	**Ascertainment of exposure (1)**	**Demonstration that outcome of interest was not present at start of study (1)**	**Study controls for age and gender (or analysis separated by gender) (2)**	**Assessment of outcome (self-harm or suicidality) (1)**	**Was follow-up long enough for outcomes to occur? (1)**	**Adequacy of follow up of cohorts (1)**	**Total quality score**
**Miller et al.,** 2018 [27]	1	1	1	1	2	1	1	1	9
** Miller N et al., ** 2018 [27]	1	1	1	1	2	1	1	1	9
**Kwak et al.,** 2018 [29]	1	1	1	1	2	1	1	1	9
**Kim et al.,** 2020 [29]	1	1	1	1	2	1	1	1	9
**Galleti et al.,** **2020**	1	1	1	1	2	1	1	1	9
Woolcombe et al., 2022 [31]	1	1	1	1	2	1	1	1	9

## Results

### Systematic review

Initially, a total of 2792 abstracts were selected through a search strategy across various search engines. They were screened and reviewed for the selected inclusion and exclusion criteria. Eleven clinical studies using DOACs as anticoagulants or studies comparing patients with and without DOACs under oral surgery procedures were found (Table 3). Due to the heterogeneity of the critical parameters, aggregation of statistical data was impossible. Therefore, not a meta-analysis but a descriptive analysis of the studies obtained was conducted.

**Table 3:** Summary of study design and study characteristics

Author	No. of patients	Mean age (range) in years	Type of DOACs used in different patients	Dental procedures	DOACs continued or discontinued prior dental procedures	Time period of DOACs discontinuation	Postoperative measures	Follow up	No. of patients with postoperative bleeding	No. of patients with delayed bleeding	Other complications	Citations
Gomez Moreno et al., 2015	57 (Rivaroxaban group + control group) Rivaroxaban group: 18 (Males: 12, Females: 6)	Rivaroxaban group: 64.4 ± 7.84 (46–73)	Rivaroxaban: 18 Without Rivaroxaban (Control): 39	Dental implants	Continued	NA	Compression with gauze soaked in tranexamic acid for 1 h and to repeat this procedure three times a day for the next 4 days	3, 8 days	NA	1 (Rivaroxaban) – The day after 2(Control) – The day after	NA	[33]
Hanken et al., 2015	337 (Rivaroxaban group Males: 34, Females: 18 Control group Males: 138, Females:147)	Rivaroxaban group: 76.7 ± 11.3 Control group 64.2 ± 10.3	Rivaroxaban: 52 Without Rivaroxaban (control):252	Dental extraction and dental implants	Continued	NA	Local compression, fibrin glue, and suturing	1, 3, 7, 10, and 14 days	2 (Control)	6(Rivaroxaban)- 3(the day after) 1(3 days) 1(5 days) 1(6 days)	NA	[26]
Miclotte et al., 2016	52 DOAC Group: 26 Control group: 26	DOAC Group: 76 Control group: 72	Rivaroxaban (69%) Dabigatran Apixaban	Dental extractions	Discontinued	15.5–48.5 h	Minor bleedings required no intervention. Moderate bleedings were managed either by contacting physician in emergency department and or through re-intervention	1,7 days	5 (DOAC group) 5 (Control group)	7 (DOAC group)	NA	[34]
Miller et al., 2018	12 (Males: 10, Females: 2)	70.6 (44–90)	Apixaban: 2 (16.7%) Dabigatran: 2 (16.7%) Rivaroxaban: 7 (58.3%) Edoxaban: 1 (8.3%)	Extractions, Alveoloplasties, Tuberosity reduction, Tori removal, Dental Implants	Among 17 surgeries, cases with -Discontinued DOACs: 9 -Continued DOACs: 1 -No records of DOACs discontinuation: 7	12–120 h	NA	NA	No records of postoperative bleeding	NA	NA	[27]
Cocero N et al., 2018	100	With comorbidities: 69 ± 10 Without comorbidities: 68.5 ± 10 (≥ 80)	Dabigatran: 21(comorbidity patients) 18 (without comorbidity) Apixaban: 25(comorbidity patients) 12 (without comorbidity) Rivaroxaban:18(comorbidity patients) 6 (without comorbidity)	Extractions	DOACs continued and a maximum of three extractions per session	NA	Compression with gauze pads, additional sutures and tranexamic acid wraps	1, 3, and 7 days	1 ( patient with comorbidity)	3( patients with comorbidities)	NA	[28]
Kwak et al., 2018	120 (Males: 67, Females: 53)	69.43 ± 11.70 (33–92)	Dabigatran: 19 Rivaroxaban: 41 Apixaban: 45 Edoxaban: 15	High bleeding risks: Scaling, curettage, extraction and implant surgery Low bleeding risks: Impression taking, root canal treatment, crown preparation and resin filling	Among 153 cases, -Discontinued DOACs: 103 -Continued DOACs: 50	1 day: 37 cases 2 days: 23 cases 3 days or longer: 10 cases	Compression hemostasis (biting down on a piece of sterilized gauze)	NA	9 (2 scaling, 3 simple extraction,3 implant surgery, and 1 resin filling)	NA	Bleeding in patients taking different drugs- Dabigatran: 1 Rivaroxaban: 5 Apixaban: 2 Edoxaban: 1	[29]
Gomez-moreno et al., 2018	71	66.7 ± 9.15 (49–71)	Dabigatran: 29 Without Dabigatran (Control): 42	Implant surgery	Continued	NA	Nonabsorbable sutures and compression with sterile gauzes soaked in 5% tranexamic acid	3, 8 days	2 (Dabigatran), 2 (Control)	NA	NA	[33]
Kim et al., 2020	64 (Males: 41, Females: 23)	74.07 ± 8.07	Rivaroxaban & Apixaban: 50 Dabigatran: 14	Apicoectomy, Periodontal Surgeries, Implants, Bone Grafting, Extractions, Other Oral and Maxillofacial Surgeries	Among 64 patients, Discontinued DOACs: 7 patients	1 day: 1 patient 2 days: 3 patients 3 days: 3 patients	Local Hemostatic agents	1 week	No records of postoperative bleeding	1	3 patients with large blood clots	[30]
Galletti et al., 2020	12 (Males: 4, Females: 8)	65.1 ± 8.57 (50–81)	Rivaroxaban: 12	Implant surgery and bone grafting	Among 12 patients, Discontinued DOACs: 12	1 day: 12 patients	Mechanical compression, tranexamic acid	1 week, 2 weeks	3	NA	4 patients suffered from swelling after surgery	[32]
Cappare et al., 2022	77 (Males: 42, Females: 35)	57.4 ± 8.57 > 20	Rivaroxaban/Apixaban: 37 Without Rivaroxaban/Apixaban (control): 40	Unilateral maxillary sinus floor elevation—lateral window approach	Continued	NA	Mechanical compression, tranexamic acid	Daily from day 1 7, 14th day, and after 1 month	4 (Rivaroxaban/Apixaban) 3 (Control)	1(Rivaroxaban/Apixaban) 0 (Control)	NA	[35]
Woolcombe et al., 2022	98 (Males: 51, Females: 47)	70 (42–91)	Rivaroxaban: 12 Apixaban:27 Edoxaban: 7 Dabigatran: 1	Routine dental extractions and surgical extractions	Among 98 patients, Discontinued: 24 Continued: 76	NA	Haemostatic packing and suturing of the socket	7 days	7 (Within 24 h)	2 (After 2 days) 1 (After 6 days) 1 (After 7 days)	NA	[31]

### Study design

Around eleven articles have been included in this review, with all studies in English. The majority of studies are retrospective cohort studies (six), [26–31] one retrospective clinical study, [32] and four case–control studies [33–35]. These studies were published in the last 10 years. Around three studies have been published between 2015 and 2016, [26, 33, 34] four studies in 2018, [27–29, 33] two in 2020, [29, 31] and the remaining two in 2022 [31, 35].

### Study characteristics

The patient sample across all eleven studies was 1,000, with a minimum of 12 and a maximum of 337 patients. The patient population consisted of 399 males and 339 females across eight studies. The mean age across all the studies was 57.4, ranging from 20 to 91 years. Rivaroxaban was the most frequently used anticoagulant DOAC type (26.5%, *n* = 265) across most studies, followed by Dabigatran and Apixaban (for details, refer to Table 3).

### Oral surgical procedures

Most of the studies obtained were principally focused on tooth extraction, especially across the seven studies. Therefore, generalizing all the data to the whole field of oral surgery is difficult. However, there were other reports on more extended oral surgical procedures, such as varied procedures like alveolplasties, tuberosity reduction, high-bleeding-risks surgeries, implant surgeries, bone grafting, maxillary sinus floor elevation, etc. (for details, see Table 3).

### DOACs

The types of DOACs used across different patients were Apixaban, Dabigatran, Rivaroxaban, and Edoxaban, as illustrated in Table 3. DOACs were mostly continued across studies prior to dental procedures, as shown in Table 3. In cases of discontinuation of DOACs prior to dental procedures across varied studies, the period of discontinuation ranged from 1–3 days.

### Test and control groups

Among eleven studies, four were case–control. In one study, Rivaroxaban was the drug of choice, with 18 cases administered with the drug and 39 without any drug. Meanwhile, Dabigatran was the DOAC drug of choice in the study, with 29 cases administered with the drug and 42 without any DOACs. Rivaroxaban and Apixaban were the drugs of choice in the remaining two studies (Table 3).

### Postoperative scenario

The postoperative measures across varied studies with different surgical procedures included compression, hemostatic agent application (mostly tranexamic acid), and sutures. In one of the studies, moderate bleeding was managed either by contacting a physician in the emergency department or through re-intervention. The patients were mainly followed up after three days or a week across the studies. The complications varied from postoperative immediate bleeding to delayed bleeding, swelling, major clots, etc., across studies (for details, see Table 3).

### Follow-up time

There was a wide variety of follow-up appointments and regimens. Whereas some authors instructed their patients to contact them after the 7th postoperative day, others conducted frequent clinical follow-up examinations after 1 day, followed by clinical examinations on the 3^rd^, 7^th^, 10^th^, and 14^th^ days, and after a month. No follow-up was reported across the two studies.

### Analysis of general outcome parameters

#### Postoperative bleeding events

In eight studies, data about postoperative local bleeding was reported. Inconsistency was reported in postoperative bleeding across all eleven studies. Among case–control studies, three reported non-stoppage of DOACs during dental extractions, dental implant surgery, and maxillary sinus elevation procedures [33, 35]. They inferred the safe administration of implant surgery and maxillary sinus floor augmentation procedures in patients taking DOACs with bare-minimum postoperative bleeding. Across the remaining six retrospective cohort studies, two exhibited no postoperative bleeding episodes [27, 30]. Overall, five studies exhibited the bare minimum of postoperative bleeding, recommending the continuation of DOACs during dental procedures [28, 31–33, 35]. The postoperative bleeding incidence was pronounced only in cases of comorbidities or complex surgical procedures [29].

#### Delayed bleeding episodes

Four studies reported no delayed bleeding [28, 30, 31, 35]. The incidence of delayed bleeding was minimal, with frequency exhibited after 2–7 days or across patients with comorbidities. However, in one study, four of the seven cases of delayed bleeding were moderate, requiring contact with a physician or a re-intervention [34].

#### DOAC vs. interruption of DOACs

The results were inconsistent and varied, with a few studies recommending DOAC administration with the bare minimum reported complications and others finding no statistically significant difference between discontinuation or continuation of drugs, especially across basic dental procedures. The reported postoperative bleeding complication was associated with comorbidities or complex dental treatments like implant placement.

## Discussion

Several studies have proposed recommendations for managing patients on DOACs during minor oral surgical procedures. However, guidelines inconsistencies have led to unnecessary discontinuation of DOACs, even in patients with a higher risk of thromboembolism. Thus, the present systematic review aims to evaluate the risk of perioperative and postoperative bleeding during minor oral surgical procedures in patients on DOACs and assist the dental professional in making an informed decision on the continuity or discontinuity of DOACs before oral surgery.

The present study included seven clinical studies that utilized DOACs as anticoagulant therapy [26–32] and four studies that compared patients on DOACs with patients without anticoagulants (control) during minor oral surgical procedures [33–35]. Of the eleven studies, six were retrospective cohort studies, one was a retrospective clinical study, and the other four were case–control studies. The mean age across all studies was 57.4, ranging from 20 to 91 years. Krammer et al., in their systematic review, referred to the term "minor oral surgery" as a variety of surgical procedures such as single tooth or multiple tooth extractions, dental implants, maxillary sinus floor augmentation, the elevation of mucoperiosteal flaps, alveoloplasties, limited oral soft tissue surgeries, and complicated osteotomies. Therefore, our review included studies with similar minor oral surgical procedures based on Krammer's criteria [6]. This systematic review showed in the studies presently included that Rivaroxaban was the most commonly used DOAC, followed by Dabigatran and Apixaban during oral surgical procedures.

Among eleven studies, five by Gomez-Moreno et al., Hanken et al., Cocero et al., Gomez-Moreno et al., and Cappare et al. continued the DOAC therapy in patients undergoing oral surgical procedures [26, 28, 33, 35]. On the contrary, six studies by Miclotte et al., Miller et al., Kwak et al., Kim et al., Galleti et al., and Woolcombe et al. either discontinued the DOACs therapy or continued it in some patients and discontinued it in others for a period varying from 12 to 120 h before the oral surgery [27, 29–32, 34]. Gomez-Moreno et al., Miller et al., and Kim et al. observed no evidence of postoperative bleeding in their studies [27, 30, 33]. Meanwhile, Hanket et al., Miclotte et al., Cocero et al., Gomez-Moreno et al., Cappare et al., Kwak et al., Galleti et al., and Woolcombe et al. reported a few patients with postoperative bleeding [26, 28, 29, 31–34]. However, the incidence of bleeding was also reported in control (individuals without DOAC therapy) groups, as mentioned in studies by Hanken et al., Miclotte et al., Gomez-Moreno et al., and Cappare et al. [26, 33–35] This suggests the invasiveness of dental procedures may also play a role in the risk of bleeding. According to a study published by Campbell et al., procedures that involve periosteal incisions may increase the risk of postoperative bleeding [36]. On the contrary, a study by Clemm et al. demonstrated that the procedure's invasiveness had no significant impact on the postoperative bleeding risk [37].

According to current Scottish Dental Clinical Effectiveness Programme (SDCEP) guidelines, patients undergoing invasive dental procedures such as extraction of 1–3 teeth or incision and drainage with a low risk of bleeding should continue their DOAC regime, which NHS recommends and most guidelines [38–40]. Conversely, patients undergoing dental procedures associated with a high risk of bleeding, such as multiple tooth extractions, flap-raising procedures, biopsies, and gingival re-contouring, should miss or delay their morning DOAC dose on the day of treatment to reduce the high risk of bleeding [41]. Besides, dentists prefer to undertake the procedure when peak DOAC concentrations have subsided, i.e., 4–6 h after the last dose. Among the studies that reported postoperative bleeding, a study by Hanken et al. reported 52 oral procedures performed under anticoagulant therapy with Rivaroxaban. The postoperative bleeding complications of these procedures were compared with those of 285 oral procedures in healthy patients without anticoagulant or antiplatelet therapy. Among 52 oral procedures, two were performed under Rivaroxaban in combination with antiplatelet therapy as per the recommendation of treating cardiologists. Although dual therapy does not match our inclusion criteria, we included this study in our research because the number of patients on dual therapy was relatively low and may not have significantly impacted the study results. The authors concluded that postoperative bleeding complications after oral surgical procedures occurred more frequently in patients receiving DOAC therapy than in healthy patients. However, the bleeding was manageable [26]. Another study by Miclotte et al. compared the difference in postoperative bleeding between 26 patients on DOACs and 26 healthy patients without any anticoagulant therapy undergoing dental extractions. DOAC therapy was discontinued for these patients on the day of the procedure. The study's results reported no significant difference in bleeding events between groups. However, patients on DOACs reported a high bleeding rate due to increased delayed bleeding [34].

Another study by Cocero et al. involved 100 patients on DOACs (Dabigatran, Apixaban, and Rivaroxaban) who underwent tooth extractions. DOAC therapy continued in these patients. Of the 100 patients, 36 patients without any comorbidities reported no bleeding [28]. Among the remaining 64 patients with comorbidities, one reported moderate bleeding, and three reported mild delayed bleeding. Delayed bleeding was attributed to the intake of one or two doses of DOACs. The authors concluded that postoperative bleeding was mainly observed in patients with comorbidities and patients who underwent extraction of multiple adjoining multi-rooted teeth.

Another study by Gomez Moreno et al. included 71 patients for dental implant placement. Of 71 patients, 29 were on DOAC therapy (the Dabigatran test group), and 42 were healthy subjects (the control group) [33]. The DOAC therapy was not interrupted during the treatment. This study reported two patients in each group with postoperative bleeding. Cappare et al. conducted a study on 77 patients involving maxillary sinus elevation procedures [35]. Among 77 patients, 37 on DOAC therapy (Rivaroxaban/Apixaban) were included as the test group, and the remaining 40 were healthy subjects (the control group). The authors continued the DOAC therapy in the test group during the procedure. The authors reported four patients in the test group and three in the control group with postoperative bleeding. In addition, a single case of delayed bleeding was also evident in the test group. Postoperative bleeding was easily managed using local measures. Both Moreno et al. and Cappare et al. concluded that no statistically significant difference in postoperative bleeding was evident in each group [33, 35].

Kwak et al. conducted a study in 120 patients (153 cases) on DOAC therapy (Dabigatran, Rivaroxaban, Apixaban, and Edoxaban) that included performing both high bleeding risk (e.g., scaling, curettage, extraction, and implant surgery) and low bleeding risk (e.g., impression taking, root canal treatment, crown preparation, and resin filling) dental procedures [29]. Of the 153 cases performed, DOAC therapy was discontinued in 103 cases and continued in 50. Postoperative bleeding was reported in nine cases (2 scaling, three simple extractions, three implant surgeries, and one resin filling), mainly categorized as high-bleeding-risk dental procedures. Thus, the authors recommended at least 24 h of discontinuation of DOAC therapy in patients undergoing high-bleeding-risk procedures, especially implant surgeries. The incidence of postoperative bleeding in this study was 5.8% (9 of 153 cases), lower than the rate reported by Mauprivez et al. and Levy et al., who reported ~ 16% postoperative bleeding. In another study by Galleti et al., 12 patients on DOACs (Rivaroxaban) underwent dental implant and prosthetic rehabilitation procedures [32]. Patients were asked to interrupt their daily dose of Rivaroxaban one day before the treatment. Of 12 patients, three (25%) reported minor bleeding after the procedure, which was managed using local hemostatic measures. The study concluded that multiple implant placements could be performed without significant bleeding complications with the discontinuation of DOACs before the surgery.

Lastly, a study by Woolcombe et al. conducted a study that included 98 patients on DOAC therapy (Rivaroxaban, Apixaban, Edoxaban, and Dabigatran) who underwent 119 dentoalveolar procedures in adherence with the Scottish Dental Clinical Effectiveness Programme (SDCEP) guidance. Postoperative bleeding was observed in 17 (14.3%) procedures [31]. Of 17 procedures, 5 (4.2%) reported minor postoperative bleeding, 1 (0.8%) required consultation in the oral surgery unit, and 11 (9.2%) procedures required the use of re-suturing, hemostatic packing, and topical use of an antifibrinolytic agent to arrest the bleeding. Bleeding in eleven patients (11 procedures) was attributed to the unsuitability of six patients for applying the specific SDCEP instructions; four patients had heart failure, which was not reported in the medical history, and the rest of the patients reported comorbidities (liver disease and kidney disease) and simultaneous intake of Clopidogrel. Although the study by Kim et al. did not experience any postoperative bleeding [30]. However, blood clots were reported in three patients, and delayed bleeding was reported in one patient. Three of the four patients were asked to discontinue DOAC therapy before surgery. Since all four complications were self-limiting, no intervention was required. In addition, four patients in the study by Galleti et al. experienced post-surgery swelling, which was manageable and subsided over time [32].

Overall, no significant complications were reported in patients on DOAC therapy who underwent minor oral surgical procedures. In some cases, postoperative bleeding was trivial and did not require any intervention [31]. Some studies required local hemostatic measures such as mechanical compression using gauze pads, additional sutures, tranexamic acid wraps, or other local hemostatic agents to arrest bleeding [26, 28, 29, 31–33, 35]. None of the study's patients required hospitalization for systemic administration of antifibrinolytics or replacement therapy. However, a study by Miclotte et al. reported moderate bleeding, managed either by contacting a physician in the emergency department or re-intervention.

Although SDCEP guidelines recommend delaying or missing the DOAC dose in procedures with a high risk of bleeding, studies by Hanken et al., Gomez-Moreno et al., Cocerno et al., Moreno et al., and Cappare et al. continued DOAC therapy in patients undergoing high-bleeding-risk procedures such as extractions, implant surgery, and maxillary sinus elevation procedures, respectively. They reported the bare minimum evidence of postoperative bleeding [26, 29, 33, 35]. Nevertheless, a sufficient gap was maintained between the last dose of DOAC and the dental procedure to minimize the risk of postoperative bleeding. Zeng et al., while comparing the effectiveness and safety outcomes of DOACs with warfarin in patients with atrial fibrillation, concluded that DOACs exerted superior effectiveness and were associated with a significant reduction in severe bleeding and intracranial hemorrhage [42]. DOACs exert their action by reversibly inhibiting the action of coagulation factors, such as thrombin or factor Xa. Since the action of DOACs is concentration-dependent. So, as soon as DOACs are eliminated from the body, the activity of the coagulation factor will be restored, minimizing the risk of excess bleeding [43].

In the present systematic review, data on the continuation or discontinuation of DOACs before dental treatment remains controversial. Some authors suggested continuing DOAC therapy in low-bleeding-risk procedures; some preferred discontinuation of DOAC therapy at least one day before the surgery; others performed the oral surgical procedures 4–6 h after the last dose of DOACs. Irrespective of the controversy, our systematic review demonstrated that minor oral surgical procedures are safe for postoperative bleeding in patients with DOAC therapy. However, dentists should decide on patients' continuity or discontinuity of DOAC therapy based on the severity of the dental procedures performed, the risk of thromboembolism, and the presence of comorbidities in the patient.

The results of our study were comparable to those of the literature review by Lanau et al., who evaluated the potential bleeding risk of DOACs and concluded that these drugs are safe to use while undergoing dental treatment [44]. However, the results of our study needed to be aligned with the results of a systematic meta-analysis by Bensi et al. [17] They concluded that DOACs have a three-times higher risk of postoperative bleeding after oral surgical procedures than anticoagulants such as warfarin or enoxaparin.

There are a few limitations to our study. For instance, only a limited number of studies fulfilled the inclusion criteria, and none were randomized controlled trials. As only a few studies were included in this review to evaluate the outcomes, the results of our study cannot be considered conclusive. Further, randomized-controlled clinical trials are required to extrapolate these results. Future directions involve the development of inexpensive antidotes that can be effective in cases of extensive bleeding.

## Conclusion

Within the limitations of this study, it can be concluded that minor oral surgical procedures are safe to perform on patients receiving DOAC therapy. In addition, postoperative bleeding can be easily managed using local measures. However, continuing or discontinuing of DOACs in patients undergoing oral surgical procedures remains controversial and requires further studies to extrapolate the results.

### Supplementary Information

**Additional file 1: Table S1.** Summary of study design and study characteristics.

## Data Availability

All data generated or analyzed during this study are included in the supplementary information file.
